# Bacterial strains isolated from river water having the ability to split alcohol ethoxylates by central fission

**DOI:** 10.1007/s11356-016-6566-8

**Published:** 2016-04-07

**Authors:** Irena Budnik, Joanna Zembrzuska, Zenon Lukaszewski

**Affiliations:** Institute of Chemistry and Technical Electrochemistry, Poznan University of Technology, pl. Sklodowskiej-Curie 5, 60-965 Poznan, Poland; Faculty of Chemical Technology, Poznan University of Technology, ul. Berdychowo 4, 60-965 Poznan, Poland

**Keywords:** Oxyethylated fatty alcohols, Biodegradation, Metabolites, LC–MS, *Citrobacter freundii*, *Enterobacter* sp., *Stenotrophomonas* sp.

## Abstract

Alcohol ethoxylates (AE) are a major component of the surfactant stream discharged into surface water. The “central fission” of AE with the formation of poly(ethylene glycols) (PEG) is considered to be the dominant biodegradation pathway. However, information as to which bacterial strains are able to perform this reaction is very limited. The aim of this work was to establish whether such an ability is unique or common, and which bacterial strains are able to split AE used as a sole source of organic carbon. Four bacterial strains were isolated from river water and were identified on the basis of phylogenetic trees as *Enterobacter strain Z2*, *Enterobacter strain Z3*, *Citrobacter freundii strain Z4,* and *Stenotrophomonas strain Z5*. Sterilized river water and “artificial sewage” were used for augmentation of the isolated bacteria. The test was performed in bottles filled with a mineral salt medium spiked with surfactant C_12_E_10_ (10 mg L^−1^) and an inoculating suspension of the investigated bacterial strain. Sequential extraction of the tested samples by ethyl acetate and chloroform was used for separation of PEG from the water matrix. LC–MS was used for PEG determination on the basis of single-ion chromatograms. All four selected and investigated bacterial strains exhibit the ability to split fatty alcohol ethoxylates with the production of PEG, which is evidence that this property is a common one rather than specific to certain bacterial strains. However, this ability increases in the sequence: *Stenotrophomonas strain Z5* < *Enterobacter strain Z2* < *Enterobacter strain Z3* = *Citrobacter freundii strain Z4*.

**Graphical Abstract:**
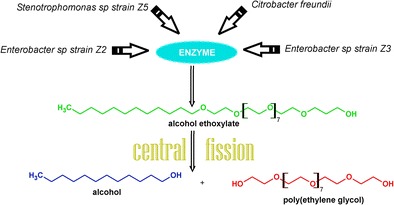
Biodegradation by central fission of alcohol ethoxylates by bacterial strains isolated from river water

Biodegradation by central fission of alcohol ethoxylates by bacterial strains isolated from river water

## Introduction

**Fig. 1:**
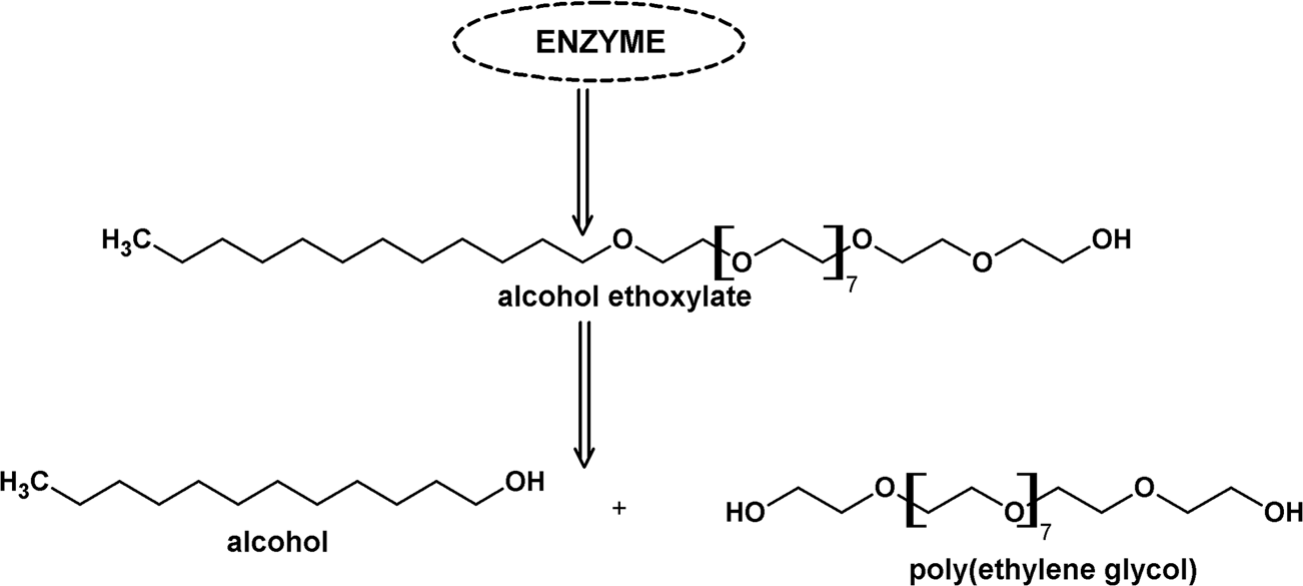
Central fission biodegradation pathway for the alcohol ethoxylates under aerobic conditions on the example of surfactant C_12_E_10_

Surfactants are a major source of anthropogenic carbon in the aquatic environment, due to their use in a huge number of household and industrial products, often typified by down-the-drain disposal. Non-ionic surfactants (NS) are a major component of the surfactant stream discharged into surface water (Zoller 1994; Fuerhacker et al. 2001; Traczyk et al. 2006; Nowicka et al. 2013a; Kopiec et al. 2014, 2015; Zembrzuska et al. 2014). 1397 million tonnes of NS were manufactured in the EC in 2012, which is more than the quantity of anionic surfactants (1201 million tonnes) (CESIO Statistics 2012). Alcohol ethoxylates (AE) are the major component of NS flux (Traverso-Soto et al. 2016). Their production in the EC in 2012 was approximately 1 million tonnes. The preferential use of AE is due to their relatively easy biodegradation under aerobic conditions. Their “central fission” with the formation of poly(ethylene glycols) (PEG) is considered to be the dominant biodegradation pathway (Fig. 1) (Patterson et al. 1967; Swisher 1987; Szymanski et al. 2000; Franska et al. 2003a). This is confirmed by identification of the oligomeric distribution and quantification of PEGs released during biodegradation (Sparham et al. 2008). PEGs are present both in water and in bottom sediments, including estuary sediments (Lara-Martín et al. 2011, 2014; Traverso-Soto et al. 2016). It should be noted that PEGs are also a technological impurity of AEs, which are a source of these compounds in the environment (0.6–10 %) (Lee et al. 2009).

The splitting of AE by bacterial consortia such as activated sludge and river water is well-documented (Patterson et al. 1967; Zanette et al. 1996; Szymanski et al. 2000; Szymański et al. 2001, 2002a; Marcomini et al. 2000; Eadsforth et al. 2006; Morrall et al. 2006). However, information as to which bacterial strains are able to perform this reaction is very limited. Bacteria of *Pseudomonas* sp. strain TR01 isolated from activated sludge at a municipal sewage treatment plant biodegrade AEs with various numbers of EO units, but not polyethylene glycols (Maki et al. 1994); however, the pathway of this degradation is not given. The gram-positive *Microbacterium* strain E19, isolated from soil, is able to split fatty alcohol ethoxylates with PEG formation (Nowicka et al. 2013b). This is the only paper concerning the central fission of AEs by an isolated bacterial strain. Investigation of this stage of AE biotransformation is significant for better understanding of the self-cleaning of the aquatic environment. Therefore, more information is required concerning which aquatic bacteria strains are able to split AEs with PEG formation. It should be remembered that several hundreds of families of microorganisms have been identified in river water (Staley et al. 2014). Thus, the question is whether such an ability is common or more specific. The aim of this study was to find an answer to this question.

River water from the River Warta (Poznan, Poland) was selected as a source of bacterial strains. This medium is in continuous contact with AEs, and bacterial consortia of river water are able to split AEs (Szymański et al. 2002a). Four bacterial strains were isolated, and all exhibited the ability to form PEG in preliminary experiments.

Sequential extraction of tested samples by ethyl acetate and chloroform was used for separation of PEG from the water matrix. This procedure has been successfully used for such a purpose (Szymański et al. 2002a, b; Nowicka et al. 2013a, b). The ethyl acetate phase contains AE, while the chloroform phase contains PEG. The LC–MS technique was selected for PEG determination on the basis of single-ion chromatograms of particular PEG homologues. Two media were selected for augmentation of the isolated bacteria: sterilized river water and “artificial sewage” used in continuous flow activated sludge tests (CFAS) (Szymanski et al. 2000). River water is a native environment for the isolated bacterial strains in terms of nutritional components. The artificial sewage contains typical components used in bacterial augmentation.

## Materials and methods

### Microorganisms

Bacterial strains were isolated from river water obtained from the River Warta in Poznan, Poland. A river water sample (100 mL) was filtered through a sterile 0.2 μm filter. The filter was incubated in agar medium at 30 °C for 24 h. Separation of pure cultures was performed using the Lisner method. Pure cultures of isolated strains were maintained on an agar medium. Four bacterial strains were isolated and were identified on the basis of phylogenetic trees as *Enterobacter* strain Z2, *Enterobacter* strain Z3, *Citrobacter freundii* strain Z4 (all three from the family *Enterobacteriaceae*), and *Stenotrophomonas* strain Z5 (family *Xanthomonadaceae*). Bacterial strains belonging to these families have been identified in many different rivers throughout the world (Ogbulie et al. 2008; Ganesan and Muthuchelian 2009; Li et al. 2014; Obukhova and Lartseva 2015).

An inoculum was prepared by suspending a loopfull from a 3 day culture in a 5 mL of a sterile enrichment broth. The solution was incubated at 30 °C for 24 h. The obtained solution was introduced into 45 mL of the sterile enrichment broth and the mixture was incubated at 30 °C for 24 h and then was introduced into 450 mL of the sterile enrichment broth and incubated at 30 °C for 72 h.

Two sterile enrichment broths were used: (i) thermally sterilized river water, and (ii) the sterile enrichment broth (artificial sewage) containing (mg L^−1^): 85 of meat extract, 15 of urea, 10 of peptone, 98 of NaHCO_3_, 14 of K_2_HPO_4_·2H_2_O, 3.5 of NaCl, 2 of CaCl_2_·2H_2_O, and 1 of MgSO_4_·7H_2_O (Zgola-Grzeskowiak et al. 2005).

### Reagents and chemicals

Polydisperse surfactant C_12_E_10_ (Sigma-Aldrich, St. Louis, MO, USA) was used as received. It is a mixture of homologues having an average of 10 oxyethylene subunits.

Chloroform, sodium chloride, and sodium hydrocarbonate of analytical grade were obtained from POCh (Gliwice, Poland). All reagents used for preparation of inoculum and mineral salt medium were from POCh, Poland, with the exception of meat extract and peptone, which were from BTL, Poland. MS-grade acetonitrile used for LC–MS measurements and the ammonium acetate used as a mobile phase additive was purchased from Sigma-Aldrich (St. Louis, MO, USA). The HPLC-grade water was prepared by reverse osmosis in a Demiwa system from Watek (Ledec nad Sazavou, Czech Republic), followed by double distillation with a quartz apparatus. Only freshly distilled water was used. All sterile solutions were obtained by treatment in an autoclave at 121 °C for 30 min.

### Biodegradation test

The test was performed in bottles filled with 200 mL of medium consisting of a mineral salt medium, spiked with surfactant C_12_E_10_ (10 mg L^−1^) and an inoculating suspension of investigated bacterial strain (5 mL). Apart from the tested surfactant, the solution contained (mg L^−1^): 33.5 of Na_2_HPO_4_·2H_2_O, 21.75 of K_2_HPO_4_, 8.5 of KH_2_PO_4_, 27.5 of CaCl_2_·2H_2_O, 22.5 of MgSO_4_·7H_2_O, 20 of NH_4_Cl, 0.25 of FeCl_3_·6H_2_O, 0.40 of MnSO_4_·H_2_O, 0.06 of H_3_BO_3_, 0.04 of ZnSO_4_·7H_2_O, and 0.035 of (NH_4_)_6_Mo_7_O_24_. The samples were left in open bottles, protected against dust and light at room temperature. All the bottles were periodically shaken on a rotary shaker to provide oxygen. The samples were preserved with 1 % formaldehyde on selected days of the biodegradation test.

### Liquid–liquid sequential extraction of substrate and PEG

15 g sodium chloride and 0.1 g sodium hydrogen carbonate were added to 50 mL of the sample. The sample was sequentially extracted with three portions of ethyl acetate, to remove NS, and with three portions of chloroform (10, 10, and 5 mL respectively) (Zgola-Grzeskowiak et al. 2008). An aliquot of the combined chloroform extracts, containing PEG, was taken for LC–MS determination, while the ethyl acetate extracts were discarded. The aliquot of chloroform extract was gently evaporated and reconstituted in the mobile phase.

### LC–MS

LC analysis was performed using the UltiMate 3000 RSLC chromatographic system from Dionex (Sunnyvale, CA, USA). 5 μL samples were injected into a 150 mm × 2.0 mm I.D. analytical column packed with 3 μm TSK Gel Amide 80 from TOSOH Bioscience (Stuttgart, Germany). The column was kept at 35 °C. The mobile phase considered of 10 mmol L^−1^ ammonium acetate in water (A) and acetonitrile (B) at a flow rate of 0.1 mL min^−1^. The following gradient was used: 0 min 99 % B, 0.5 min 50 % B, 4 min 5 % B, 7 min 1 % B, 10 min 1 % B. A pre-run time of 9 min was left before the next injection.

**Fig. 2:**
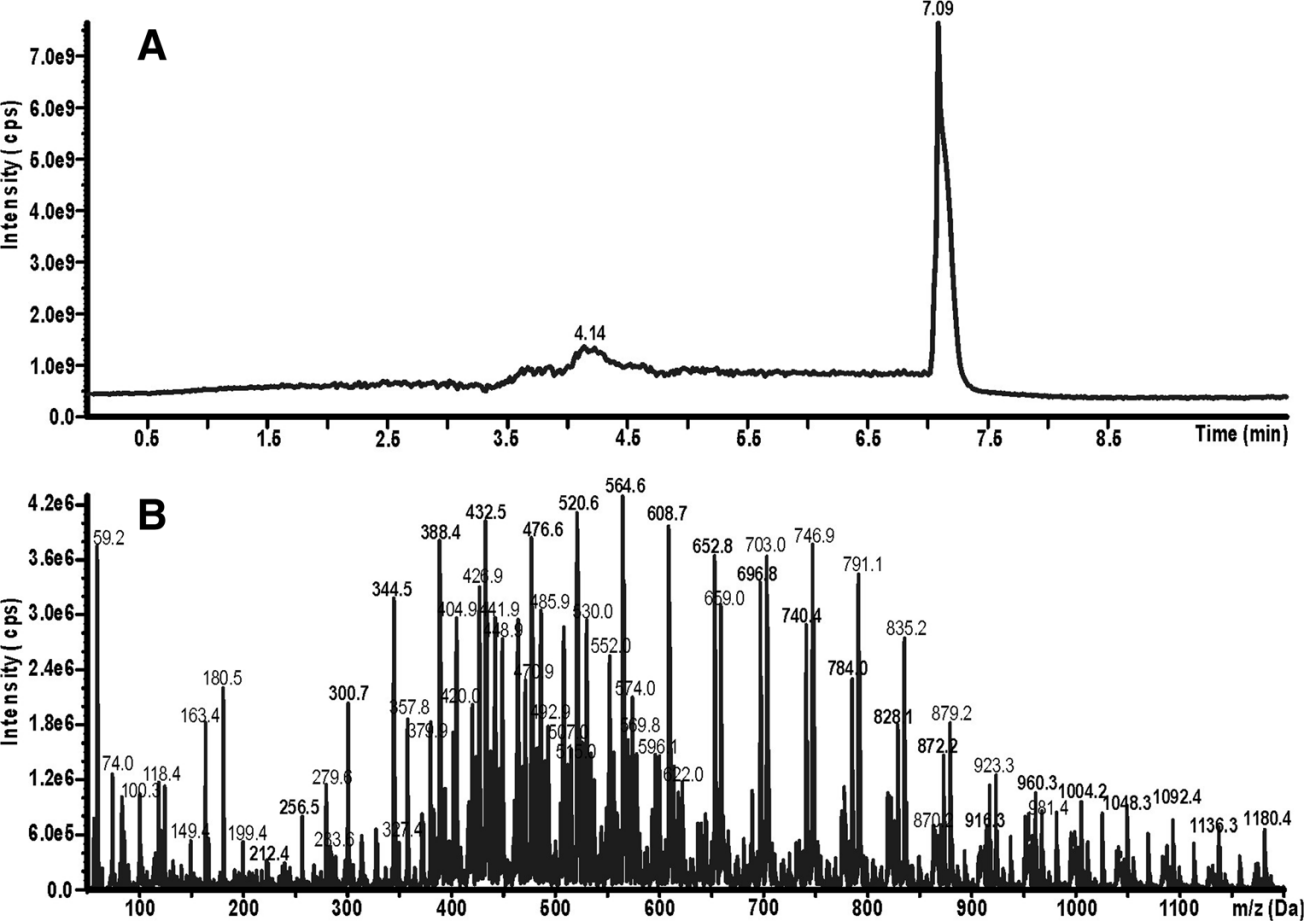
Total ion chromatogram of the chloroform extract of biodegradation test sample (**a**); and mass spectrum of peak at 7.09 min of chromatogram (**b**). The sample was obtained from seventh day of biodegradation test of surfactant C_12_E_10_ by bacteria *Enterobacter* strain Z3. The bacteria was augmented on an artificial sewage

**Fig. 3:**
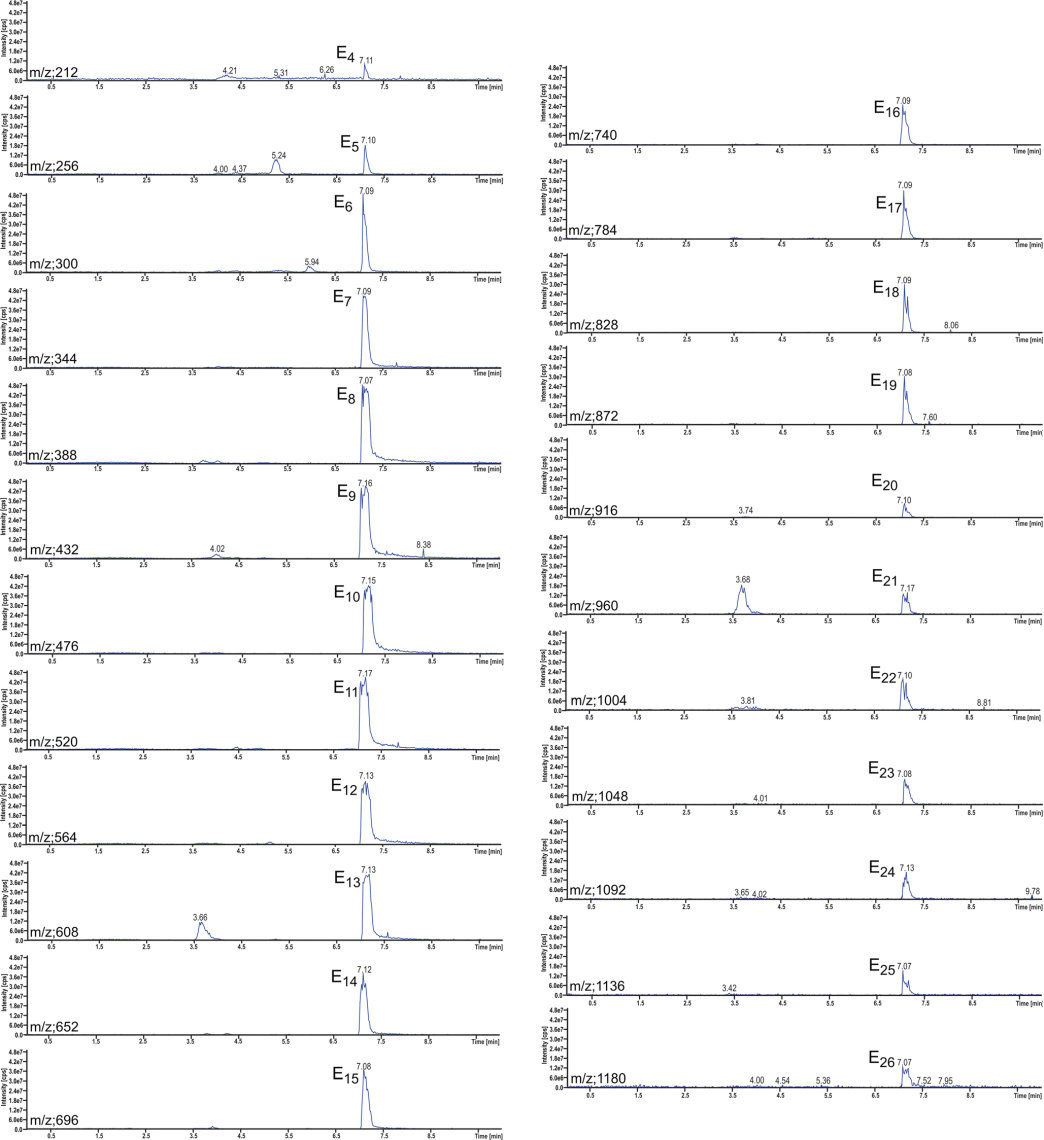
Single-ion chromatograms of particular homologues of polyethylene glycols corresponding to the sample as in Fig. 2

The LC system was connected to an API 4000 QTRAP triple quadrupole mass spectrometer from AB Sciex (Foster City, CA, USA). The Turbo Ion Spray source was operating in positive ion mode. Analytes were detected using the following settings for the ion source and mass spectrometer: curtain gas 10 psi, nebulizer gas 45 psi, auxiliary gas 45 psi, temperature 350 °C, ion spray voltage 5500 V, declustering potential 36 V, and collision gas set to medium. Total ion chromatograms within an *m*/*z* range between 100 and 1200 and mass spectra corresponding to the obtained chromatographic peaks were recorded. An example of a total ion chromatogram of chloroform extract is shown in Fig. 2a. The peak at 7.09 min corresponds to PEGs, being identified on the basis of the mass spectrum shown in Fig. 2b. PEGs are identified as a series of mass peaks which differ by *m*/*z* = 44 Da (Franska et al. 2003b), i.e., the mass of a single oxyethylene unit. A series of mass peaks having *m*/*z* = 212 + n44 corresponds to ammonium complexes of PEGs having 4 + n oxyethylene subunits. Apart from this series, another series having *m*/*z* = 658 + n44 appears, which corresponds to ammonium complexes of carboxylated surfactant C_12_E_x_; there is also a series *m*/*z* = 404 + n22 (double ammonium complexes of carboxylated surfactant C_12_E_x_). Apart from ammonium complexes, weaker signals of complexes with Na(I) and K(I) appear, which form a “forest” of lower mass peaks. Single-ion chromatograms at particular *m*/*z* values were recorded, and the areas of the peaks appearing were used as the analytical signals of determined individual PEGs. The example of a series of single-ion chromatograms of PEGs having 4–26 oxyethylene subunits is shown in Fig. 3. Areas of peaks were presumed to be equivalent to PEG concentration.

## Results and discussion

Eight parallel flask biodegradation tests were performed with bacterial strains isolated from river water. Each of the four isolated stains was tested twice: (i) with bacteria augmented in sterilized river water, and (ii) with bacteria augmented in artificial sewage. Tests were performed in aerobic conditions with surfactant C_12_E_10_ as the sole source of organic carbon. Determination of PEG concentration was performed at the very beginning of the tests and on the seventh day of the test. The samples were sequentially extracted with ethyl acetate (to remove residual C_12_E_10_) and with chloroform. Aliquots of chloroform extracts were determined for PEG concentration.

**Fig. 4:**
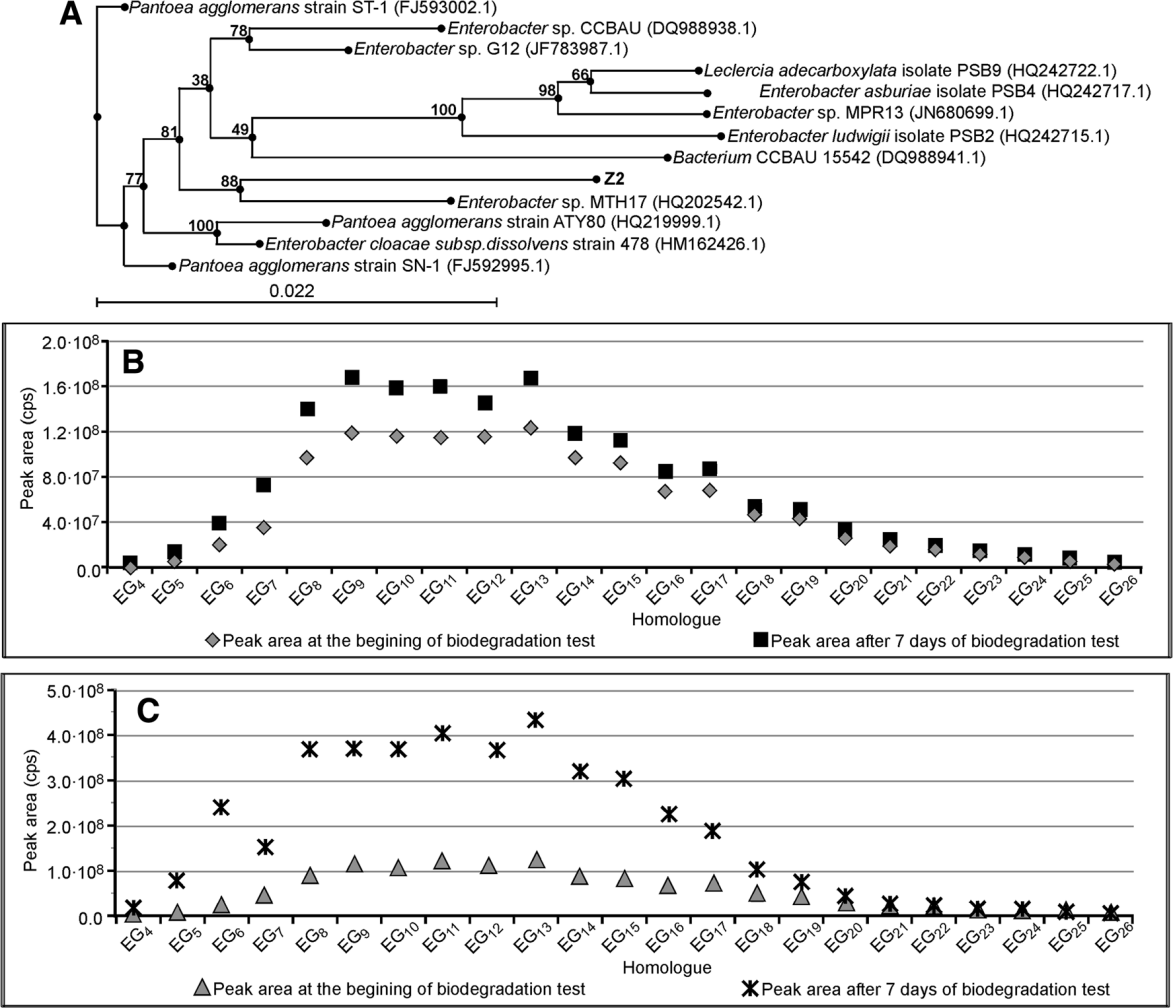
Phylogenetic tree of bacteria *Enterobacter* strain Z2 (**a**). Concentration of particular PEG homologues (represented as the *peak area*) at day seven and at the beginning of the biodegradation test of surfactant C_12_E_10_ with bacteria *Enterobacter* strain Z2. *Inoculum* was augmented on sterilized river water (**b**). As Fig. 4c with *inoculum* augmented on an artificial sewage

The results for *Enterobacter strain Z2* are shown in Fig. 4a, together with its phylogenetic tree, being the “proof of identity” of the strain. The concentration of a particular homologue is represented by a peak area on a single-ion chromatogram. The concentrations of homologues having 4–26 oxyethylene units was determined. The polydispersity of PEG obtained after the central fission of surfactant C_12_E_10_ is an obvious consequence of the polydispersity of surfactant C_12_E_10_. A strong increase in the concentration of homologues having 5–18 oxyethylene subunits is apparent in Fig. 4c. The results at the seventh day of the experiment are approximately 4 times higher than at the beginning of the test. This increase is obvious evidence of the central fission of surfactant C_12_E_10_ by bacteria of *Enterobacter strain Z2*. The presence of PEG homologues at the beginning of the test is due to their presence in surfactant C_12_E_10_ as a technological impurity. A lower increase in PEG concentration is also observed in Fig. 4b, i.e., in the test with *Enterobacter strain Z2* augmented in sterilized river water. It seems that this medium is less favorable to the growth of *Enterobacter strain Z2* than the artificial sewage.

**Fig. 5:**
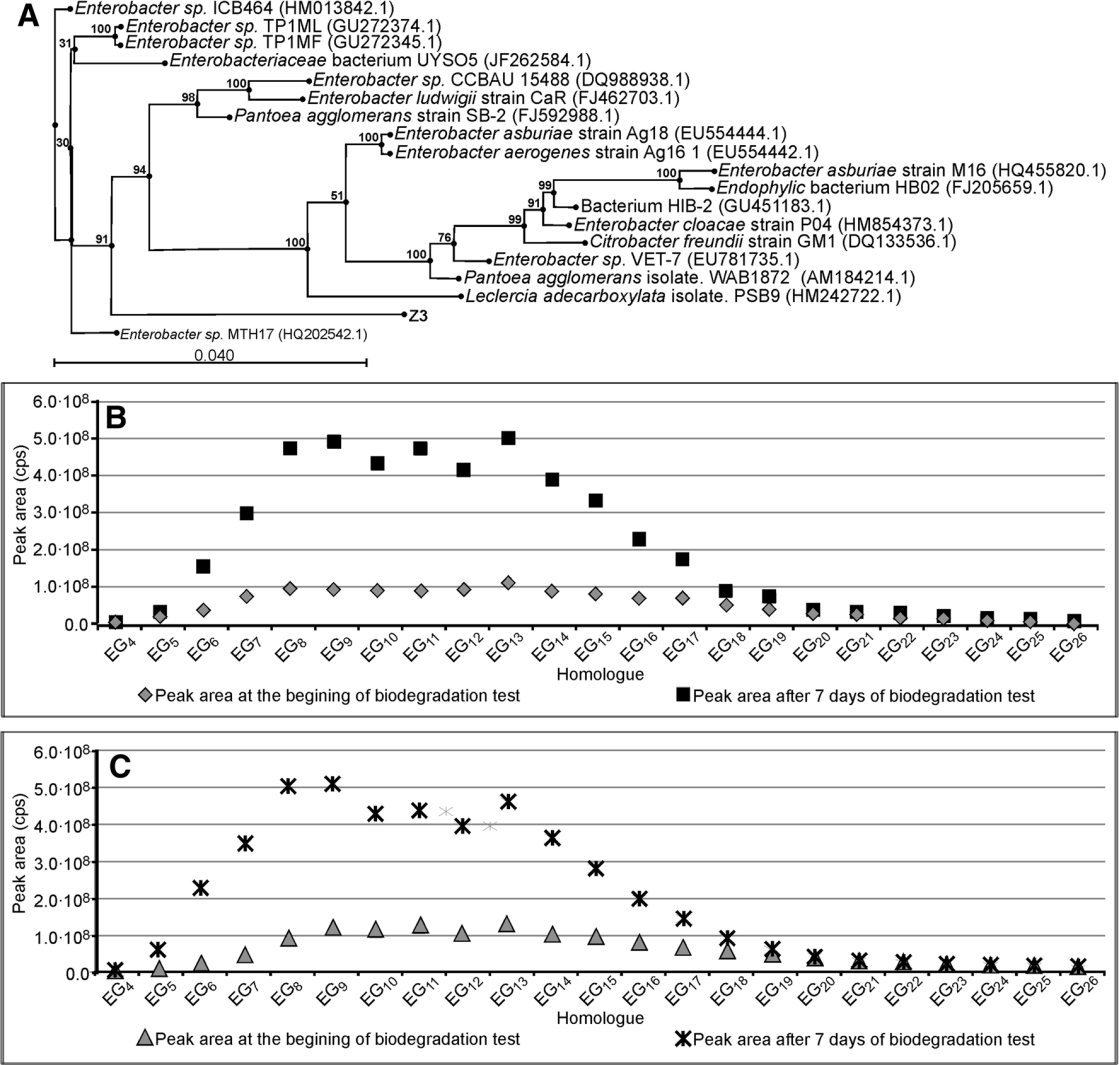
Phylogenetic tree of bacteria *Enterobacter* strain Z3 (**a**). Concentration of particular PEG homologues (represented as the *peak area*) at day seven and at the beginning of the biodegradation test of surfactant C_12_E_10_ with bacteria *Enterobacter* strain Z3. *Inoculum* was augmented on sterilized river water (**b**). As Fig. 5c with *inoculum* augmented on an artificial sewage

**Fig. 6:**
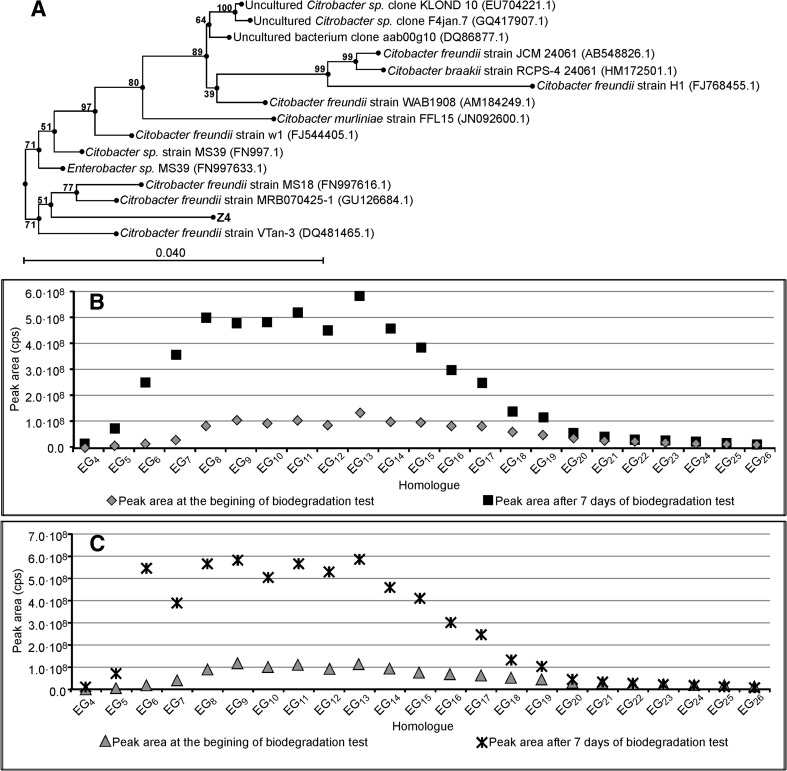
Phylogenetic tree of bacteria *Citrobacter freundii* strain Z4 (**a**). Concentration of particular PEG homologues (represented as the peak area) at day seven and at the beginning of the biodegradation test of surfactant C_12_E_10_ with bacteria *Citrobacter freundii* strain Z4. *Inoculum* was augmented on sterilized river water (**b**). As Fig. 6c with *inoculum* augmented on an artificial sewage

**Fig. 7:**
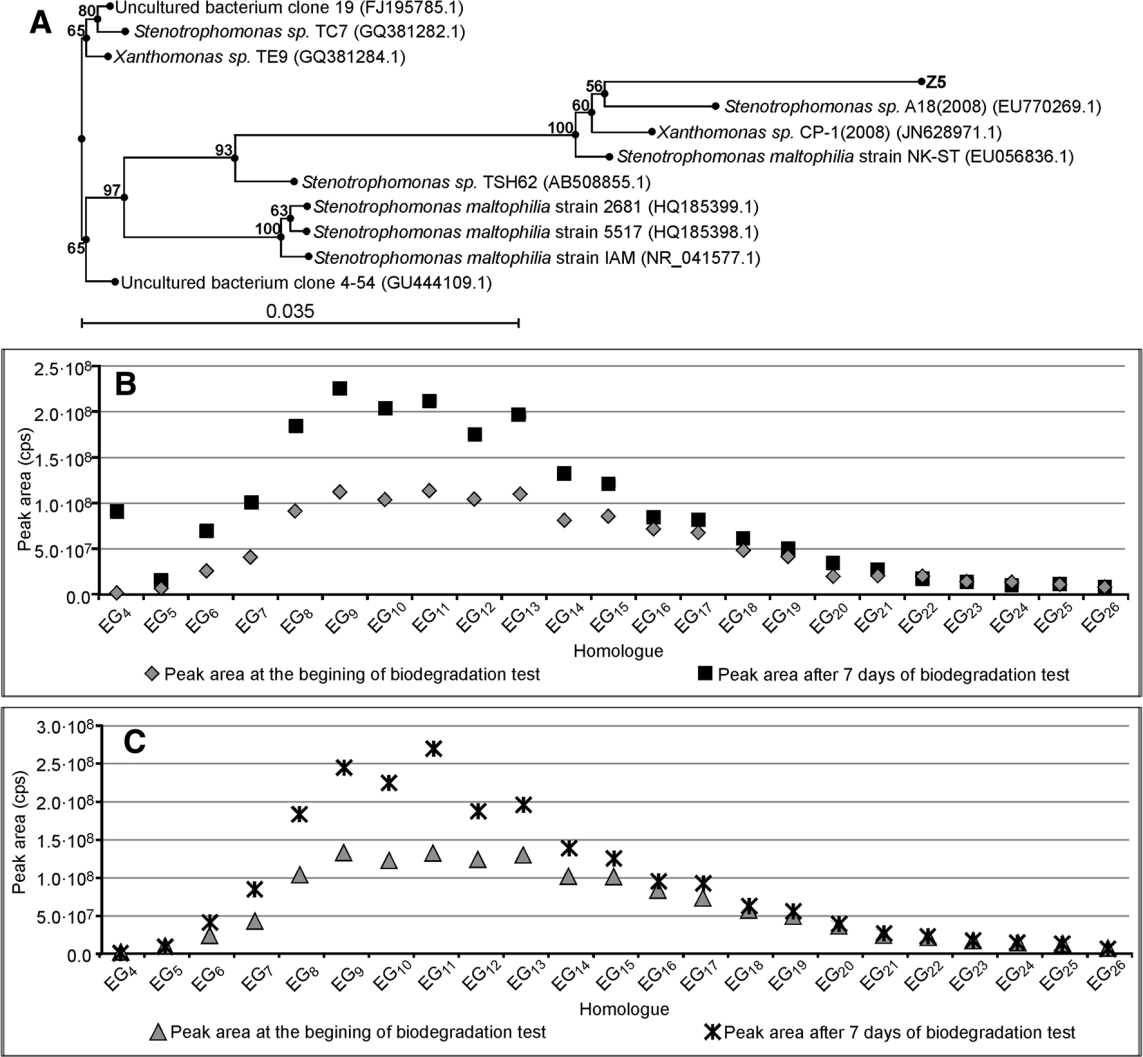
Phylogenetic tree of bacteria *Stenotrphomonas* strain Z5 (**a**). Concentration of particular PEG homologues (represented as the peak area) at day seven and at the beginning of the biodegradation test of surfactant C_12_E_10_ with bacteria *Stenotrphomonas* strain Z5. *Inoculum* was augmented on sterilized river water (**b**). As Fig. 7c with *inoculum* augmented on an artificial sewage

The results concerning *Enterobacter strain Z3*, *Citrobacter freundi strain Z4,* and *Stenotrophomonas strain Z5*, together with their phylogenetic trees, are shown in Figs. 5, 6, and 7 respectively. All these bacterial strains causes an increase in PEG concentration, although to different degrees. *Enterobacter strain Z3* (Fig. 5) and *Citrobacter freundii strain Z4* (Fig. 6) show a stronger ability to split surfactant C_12_E_10_ with PEGs formation. In both cases the PEG concentration is 5 times higher than that at the start of the biodegradation tests. No significant difference is observed between the experiments with bacteria augmented with sterilized river water and with the artificial sewage. The production of PEG by *Stenotrophomonas strain Z5* is much weaker (Fig. 7). The results on the seventh day of the test are only 2–2.5 times higher than at the start of the biodegradation tests.

The fact that all investigated bacterial strains exhibit the ability to split fatty alcohol ethoxylate with the production of PEGs is evidence that such a property is a common one rather than specific to certain bacterial strains. This conclusion may be supported by the splitting of fatty alcohol ethoxylates with PEG formation by bacteria of *Microbacterium* strain E19 (Nowicka et al. 2013b). This bacterium is gram-positive (belonging to the phylum Actinobacteria), while all four strains investigated in the present work are gram-negative (belonging to the phylum Proteabacteria). Moreover, they belong to two different families: Enterobacteriaceae (*Enterobacter* strain Z2, *Enterobacter* strain Z3, *Citrobacter freundi* strain Z4) and Xanthomonadaceae (*Stenotrophomonas* strain Z5*)*. Thus, the ability to split AEs in accordance with the central fission pathway is a common property of the kingdom Bacteria. However, different strains exhibit variable PEG productivity; even two strains belonging to the same genus *Enterobacter* (strains Z2 and Z3) show significantly different PEG productivity. Moreover, the conditions of bacterial augmentation have a strong effect on the AE central fission. Finally, the presence of carboxylated AEs in mass spectra (see Fig. 2b) may be evidence of the existence of an alternative AE biodegradation pathway, parallel to central fission.

## Conclusions

The ability to split alcohol ethoxylates in accordance with the central fission pathway with the formation of poly(ethylene glycols) is a common property of microorganisms belonging to the kingdom *Bacteria*. Polydisperse PEGs are produced from polydisperse AEs. This ability is subject to variation among strains belonging to various families. Even strains belonging to the same family exhibit differences in their PEG productivity. The conditions of bacterial augmentation are also significant for the PEG productivity of particular strains.
